# Effect of galcanezumab on severity and symptoms of migraine in phase 3 trials in patients with episodic or chronic migraine

**DOI:** 10.1186/s10194-021-01215-9

**Published:** 2021-02-06

**Authors:** Michael Ament, Kathleen Day, Virginia L Stauffer, Vladimir Skljarevski, Mallikarjuna Rettiganti, Eric Pearlman, Sheena K. Aurora

**Affiliations:** 1 Ament Headache Center, 80206 Denver, CO USA; 2 Eli Lilly and Company, 46285 Indianapolis, IN USA

**Keywords:** Galcanezumab, Migraine, Episodic, Chronic, Symptoms

## Abstract

**Background:**

Galcanezumab, a humanized monoclonal antibody that binds calcitonin gene-related peptide, has demonstrated a significant reduction in monthly migraine headache days compared with placebo. Here, we analyze data from 3 randomized clinical trials (2 episodic trials [EVOLVE-1, EVOLVE-2] and 1 chronic trial [REGAIN]), to examine if galcanezumab also alleviates the severity and symptoms of migraine.

**Methods:**

The episodic migraine trials were 6-month, double-blind studies in patients with episodic migraine (4–14 monthly migraine headache days). The chronic migraine trial was a 3-month, double-blind study in patients with chronic migraine (≥ 15 headache days per month, where ≥ 8 met criteria for migraine). Patients (18–65 years) were randomized to placebo or galcanezumab 120 mg with a 240-mg loading dose or 240 mg. Patients recorded headache characteristics, duration, severity, and presence of associated symptoms with each headache. The outcomes analyzed were changes from baseline in number of monthly migraine headache days with nausea and/or vomiting, photophobia and phonophobia, aura, and prodromal symptoms other than aura. Additional outcomes analyzed included the number of moderate-to-severe monthly migraine headache days, number of severe migraine headache days, and mean severity of remaining migraine headache days. Change from baseline in the proportion of days with nausea and/or vomiting and the proportion of days with photophobia and phonophobia among the remaining monthly migraine headache days were also analyzed.

**Results:**

Galcanezumab was superior to placebo in reducing the frequency of migraine headache days with associated symptoms of migraine such as nausea and/or vomiting, photophobia and phonophobia, and prodromal symptoms. Galcanezumab reduced the frequency of migraine headache days with aura in the episodic migraine studies. There was a significant reduction in the proportion of remaining migraine headache days with nausea and/or vomiting for the episodic and chronic migraine studies, and with photophobia and phonophobia for the episodic migraine studies. Galcanezumab was superior to placebo in reducing the number of monthly moderate-to-severe migraine headache days and the overall and monthly severe migraine headache days.

**Conclusions:**

Galcanezumab reduces the frequency of migraine headache days and can alleviate potentially disabling non-pain symptoms on days when migraine is present in patients with episodic or chronic migraine.

**Trial registration:**

NCT, NCT02614183 (EVOLVE-1), registered 25 November 2015; NCT, NCT02614196, (EVOLVE-2), registered 25 November 2015; NCT, NCT02614261 (REGAIN), registered 25 November 2015.

## Background

According to the Global Burden of Disease Study 2016, migraine is the second leading cause of years lived with disability worldwide, after low back pain, and represents 45.1 million (95 % uncertainty interval [UI]: 29.0–62.8 million) years lived with disability [1]. The global age-standardized prevalence of migraine, 18.9 % (18.1–19.7) for women and 9.8 % (9.4–10.2) for men, represents a worldwide total of 1.04 billion (95 % UI: 1.00-1.09 billion) individuals with migraine [2]. Despite its wide prevalence, migraine is often under-treated and inadequately recognized [3, 4].

Migraine is primarily defined by its most prominent symptom which is a unilateral, moderate-to-severe, pulsating headache lasting from 4 to 72 hours and aggravated by routine physical activity usually accompanied by nausea and/or vomiting or photophobia and phonophobia [5]. Many patients experience a prodromal or premonitory phase that may begin hours or days before the headache phase of a migraine attack [5] and some patients experience a postdromal phase following headache resolution. These premonitory symptoms may include fatigue, difficulty concentrating, neck stiffness, sensitivity to light and/or sound, nausea, blurred vision, yawning, and pallor [5–8]. Approximately 20–30 % of patients may also experience aura (commonly manifested as visual disturbances) usually beginning approximately 60 minutes before the headache phase of a migraine attack [9, 10].

Calcitonin gene-related peptide (CGRP) monoclonal antibodies are efficacious in the treatment of migraine [11–13]. Phase 3, double-blind, placebo-controlled studies have shown that the CGRP monoclonal antibodies erenumab [7, 14, 15], fremanezumab [16, 17], galcanezumab [18–20], and eptinezumab [21, 22], now approved in the United States and other countries, are efficacious in decreasing monthly migraine headache day frequency [23]. A phase 2 study of fremanezumab also showed reductions in migraine-associated symptoms of nausea or vomiting, photophobia, and phonophobia in patients with episodic migraine [24]. However, there is minimal information currently available from phase 3 studies on how these compounds further affect the severity of the remaining migraine headache days and the associated symptoms that are often very disabling to the patient.

Here, we examine the efficacy of galcanezumab on the severity and symptoms of migraine in patients with episodic and chronic migraine, and we assess if galcanezumab leads to reductions in non-pain symptoms associated with migraine such as nausea and vomiting, phonophobia and photophobia, aura, and prodromal symptoms.

## Methods

This investigation, a post-hoc analysis of results from three phase 3, randomized, double-blind, placebo-controlled clinical trials, examines the effects of galcanezumab on the severity and symptoms of migraine in patients with episodic and chronic migraine.

In these studies, there were a total of 2289 patients with either episodic migraine (N = 1176 in EVOLVE-1 [NCT02614183] and EVOLVE-2 [NCT02614196]) or chronic migraine (N = 1113 in REGAIN [NCT02614261]), with or without aura. All patients gave written informed consent to participate in the study. The studies were conducted with the approval of the independent Ethics Committees of the participating institutes and in accordance with the Declaration of Helsinki, the International Conference on Harmonization Good Clinical Practice guidelines, and local regulations.

Data from the two episodic migraine studies were pooled and the chronic migraine study was analyzed separately. The two episodic migraine studies (EVOLVE-1 [18] and EVOLVE-2 [19]) examined whether galcanezumab at doses of 120 mg per month with a 240-mg loading dose or 240 mg per month was superior to placebo in the preventive treatment of episodic migraine. The chronic study, REGAIN [20], was designed to determine if galcanezumab was superior to placebo in the preventive treatment of chronic migraine at doses of 120 mg per month with a 240-mg loading dose or 240 mg per month. Details of the study designs of the trials are shown in the previously published manuscripts for these clinical trials [18–20].

Patients were between the ages of 18 and 65 years, with a diagnosis of migraine with or without aura, as defined by International Headache Society (IHS) International Classification of Headache Disorders (ICHD) -3rd edition, beta version [25] for at least 1 year prior to enrollment and with migraine onset prior to 50 years of age. Patients in the episodic migraine studies needed to have 4 to 14 migraine headache days with an average of no less than 2 migraine attacks per month during the previous 3 months, and those in the chronic migraine study needed to have at least 15 headache days per month, of which 8 or more had migraine features, with at least 1 or more headache-free days per month for the past 3 months.

Patients were excluded from the study if they were enrolled in, or had participated in, a clinical trial involving an investigational product within the last 30 days, if they had current or prior exposure to galcanezumab or another CGRP antibody within the past 12 months, or if they failed to have an efficacy response to 3 or more classes (level A or B evidence) of migraine preventive treatments [26]. Patients with a history or presence of any other medical illness (including acute and/or serious cardiovascular events), significant laboratory abnormality, or who used opioids or barbiturate-containing analgesics more than twice per month in more than 2 of the past 6 months were also excluded, as were patients diagnosed with medication overuse headache (episodic studies only). Patients who had a history of ≥ 15 migraine headache days per month (i.e., chronic migraine) were excluded from the episodic migraine studies.

Patients recorded headache characteristics, duration, and severity, as well as presence of nausea, vomiting, photophobia, phonophobia, aura, and prodromal symptoms other than aura with each headache in a daily electronic patient-reported outcome (ePRO) diary. The prodromal symptoms were not specified in our studies. Per the protocol patients had to answer with yes/no for prodromal symptoms other than aura in the eDiary. Migraine headache days and headache days were derived using all headache information recorded in the ePRO diary. A migraine headache day was defined as a calendar day on which a migraine or probable migraine headache occurred and remaining migraine headache days refer to days in which a migraine or probably migraine occurred during the study treatment periods (i.e., those that still remain after study treatment). Rating for migraine severity was 1 = mild, 2 = moderate, and 3 = severe.

### Statistical analyses

Baseline demographics and disease characteristics were summarized using appropriate summary statistics such as mean and standard deviation for continuous variables, and frequency and percentages for categorical variables. The change from baseline in continuous repeated measures outcomes was analyzed using a mixed model repeated measures analysis (MMRM) with an unstructured covariance structure to account for the correlation among outcomes measured on the same subject. All models included the following variables: baseline value, treatment group, month, pooled region/country, treatment by month interaction, and baseline by month interaction. All models involving the pooled episodic migraine studies included an additional study variable which identified the two episodic studies.

The outcomes analyzed were changes from baseline in number of monthly migraine headache days with nausea and/or vomiting, photophobia and phonophobia, aura, and prodromal symptoms other than aura, number of moderate-to-severe migraine headache days, number of severe migraine headache days, and mean severity of remaining migraine headache days. Additionally, among the remaining monthly migraine headache days, change from baseline in the proportion of days with nausea and/or vomiting, and the proportion of days with photophobia and phonophobia were also analyzed.

Treatment effects from the MMRM models are presented using least squares (LS) means for the overall treatment effect, and the difference in LS means compared with placebo and corresponding 95 % confidence intervals (CI). All statistical tests were two-sided at the significance level of 5 %. No adjustments for multiple testing or multiple comparisons were done and thus results should be considered exploratory. All statistical analyses were performed using SAS® Enterprise Guide. SAS and all other SAS Institute Inc. product or service names are registered trademarks or trademarks of SAS Institute Inc., Cary, NC, USA.

## Results

### Baseline characteristics

Overall, baseline demographic and disease characteristics were generally consistent between the groups in the episodic migraine studies and between the groups in the chronic migraine study with the exception of migraine headache day frequency and related symptoms (Table 1).

**Table 1:** Baseline demographics and disease characteristics

Variable	EVOLVE-1 and EVOLVE-2 (Episodic Migraine Trials)				REGAIN (Chronic Migraine Trial)			
	PBO N = 894	GMB 120 mg N = 444	GMB 240 mg N = 435	Total N = 1773	PBO N = 558	GMB 120 mg N = 278	GMB 240 mg N = 277	Total N = 1113
Age, years, mean (SD)	41.5 (11.3)	40.6 (11.4)	40.1 (11.5)	41.0 (11.4)	41.6 (12.1)	39.7 (11.9)*	41.1 (12.4)	41.0 (12.1)
Female, n (%)	755 (84.5)	378 (85.1)	366 (84.1)	1499 (84.6)	483 (87)	237 (85)	226 (82)	946 (85.0)
Years since migraine diagnosis, mean (SD)	20.3 (12.3)	20.7 (12.1)	19.5 (11.9)	20.2 (12.2)	21.9 (12.9)	20.4 (12.7)	20.1 (12.7)*	21.1 (12.8)*
Number of migraine headache days/month	10.9 (2.0)	10.9 (2.0)	10.7 (2.0)	10.8 (2.0)	19.6 (4.6)	19.4 (4.3)	19.2 (4.6)	19.4 (4.5)
Number of severe migraine headache days/month	2.6 (2.4)	2.5 (2.3)	2.7 (2.3)	2.6 (2.3)	6.6 (5.2)	6.7 (4.9)	6.6 (4.8)	6.6 (5.0)
Number of moderate-severe migraine headache days	7.4 (3.0)	7.4 (3.0)	7.5 (3.1)	7.4 (3.1)	16.1 (5.4)	16.0 (5.2)	15.7 (5.4)	16.0 (5.4)
Mean severity of migraine headache days^a^	2.1 (0.4)	2.1 (0.4)	2.1 (0.4)	2.1 (0.4)	2.2 (0.4)	2.2 (0.4)	2.2 (0.4)	2.2 (0.4)
Number of migraine headache days with aura	3.1 (3.8)	2.8 (3.4)	3.1 (3.6)	3.0 (3.7)	5.0 (6.9)	5.3 (7.3)	4.8 (7.0)	5.0 (7.0)
Number of migraine headache days with nausea and/or vomiting	4.6 (3.4)	4.7 (3.4)	4.8 (3.3)	4.7 (3.4)	9.2 (6.9)	8.8 (6.3)	8.1 (6.5)*	8.8 (6.7)
Number of migraine headache days with photophobia and phonophobia	8.4 (3.5)	8.1 (3.6)	8.1 (3.6)	8.3 (3.5)	15.2 (7.2)	14.8 (7.0)	14.0 (7.6)*	14.8 (7.3)
Number of migraine headache days with prodromal symptoms	3.9 (3.8)	3.8 (3.8)	3.7 (3.7)	3.9 (3.8)	6.8 (7.3)	7.3 (7.5)	7.1 (7.3)	7.0 (7.4)
Proportion of migraine headache days with nausea and vomiting	40.4 (30.1)	41.8 (32.0)	42.3 (30.2)	41.2 (30.6)	46.4 (31.3)	44.7 (29.3)	41.5 (30.3)	44.8 (30.6)
Proportion of migraine headache days with photophobia and phonophobia	74.5 (30.2)	72.8 (31.8)	73.9 (30.7)	73.9 (30.7)	76.4 (29.6)	75.0 (29.7)	71.6 (32.7)	74.8 (30.5)

*p ≤ 0.05 vs. PBO; **p ≤ 0.01 vs. PBO. ^a^Severity ratings: 1 = mild, 2 = moderate, 3 = severe

*GMB* Galcanezumab, *PBO *Placebo, *SD *Standard deviation

### Efficacy

#### Symptoms - migraine headache days with nausea and/or vomiting, photophobia and phonophobia, prodrome, and aura

### Migraine headache days with nausea and/or vomiting

**Fig. 1:**
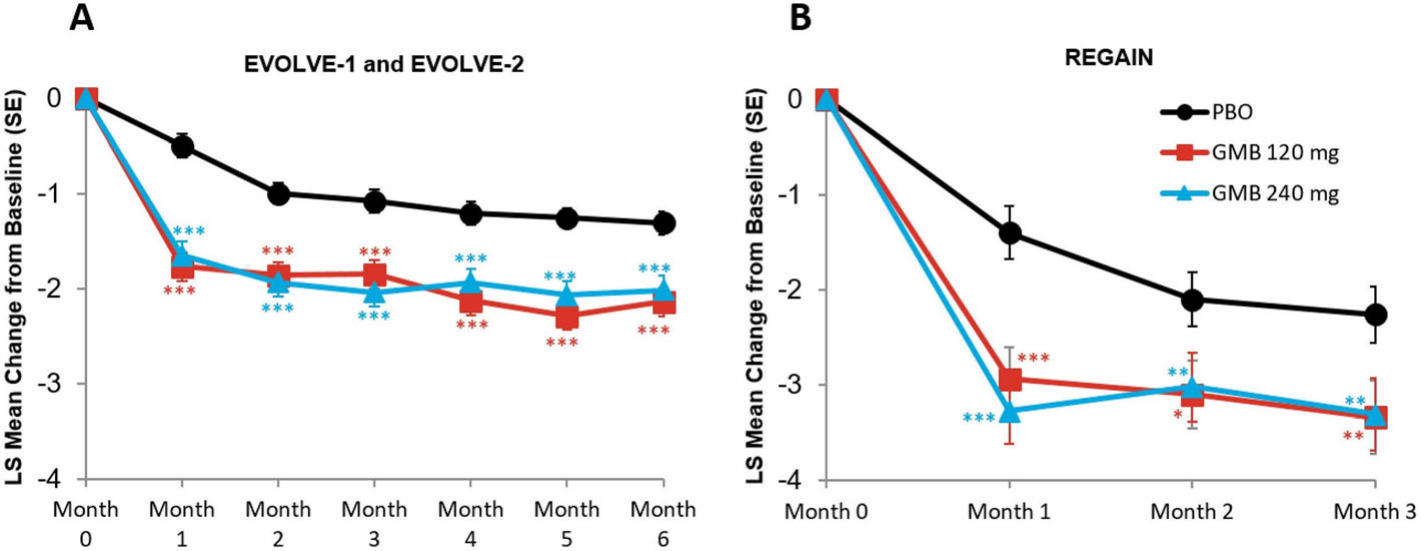
Monthly mean change in monthly migraine headache days with nausea and/or vomiting. *p≤0.05 vs PBO; **p≤0.01 vs PBO; ***p≤0.001 vs PBO. GMB, galcanezumab; LS,least squares; PBO, placebo; SE, standard error

The monthly mean change in monthly migraine headache days with nausea and/or vomiting are presented in Fig. 1A and B. In the episodic migraine studies, mean reduction from baseline in the number of monthly migraine headache days with nausea or vomiting across all 6 months was 2.0 days for galcanezumab 120 mg, 1.9 days for galcanezumab 240 mg, and 1.1 days for placebo. The mean differences versus placebo were statistically significant for both treatment groups (for 120 mg, mean difference = -0.95 days, 95 % CI, -1.19 to -0.70, *p* < 0.001; for 240 mg, mean difference = -0.88 days, 95 % CI, -1.13 to -0.63, *p* < 0.001). In the chronic migraine study, mean reduction from baseline in the number of monthly migraine headache days with nausea or vomiting across all 3 months was 3.1 days for galcanezumab 120 mg, 3.2 days for galcanezumab 240 mg, and 1.9 days for placebo. The mean differences versus placebo were statistically significant for both treatment groups (for 120 mg, mean difference = -1.21 days, 95 % CI, 1.82 to -0.59, *p* < 0.001; for 240 mg, mean difference = -1.28 days, 95 % CI, -1.90 to -0.66, *p* < 0.001).

#### Migraine headache days with photophobia and phonophobia

**Fig. 2:**
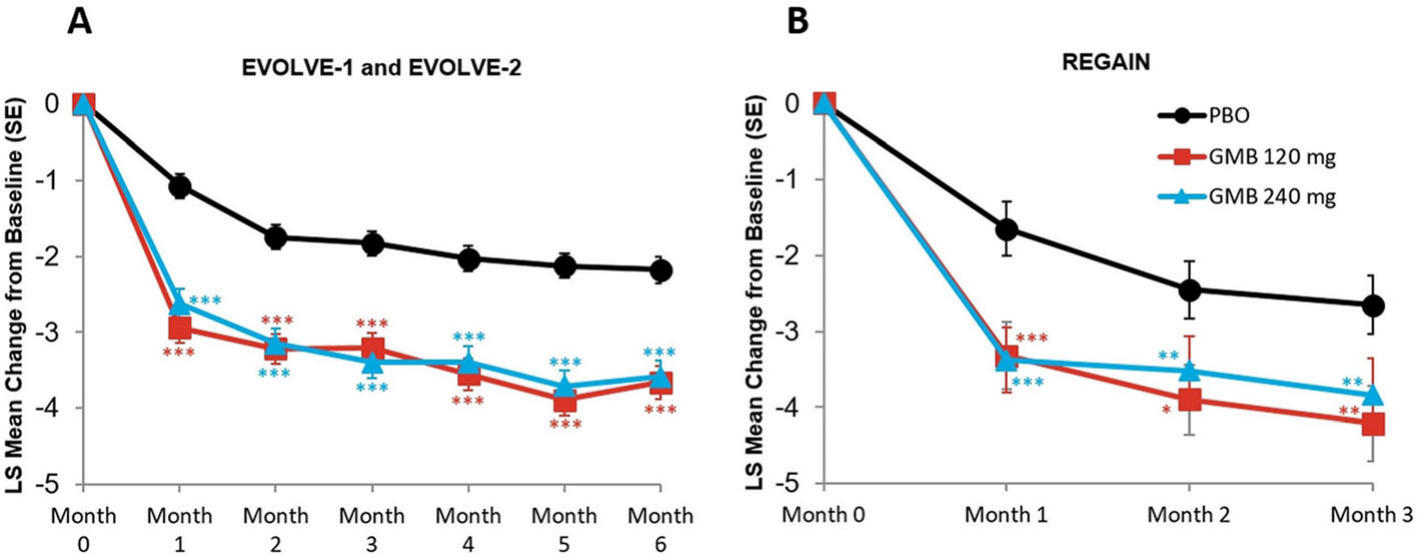
Monthly mean change in monthly migraine headache days with photophobia and phonophobia. *p≤0.05 vs PBO; **p≤0.01 vs PBO; ***p≤0.001 vs PBO. GMB, galcanezumab; LS,least squares; PBO, placebo; SE, standard error

The monthly mean change in monthly migraine headache days with photophobia and phonophobia are presented in Fig. 2A and B. In the episodic migraine studies, mean reduction from baseline in the number of monthly migraine headache days with photophobia and phonophobia across all 6 months was 3.4 days for galcanezumab 120 mg, 3.3 days for galcanezumab 240 mg, and 1.8 days for placebo. The mean differences versus placebo were statistically significant for both treatment groups (for 120 mg, mean difference = -1.58 days, 95 % CI, -1.93 to -1.23, *p* < 0.001; for 240 mg, mean difference = -1.48 days, 95 % CI, -1.83 to -1.13, *p* < 0.001). In the chronic migraine study, mean reduction from baseline in the number of monthly migraine headache days with photophobia and phonophobia across all 3 months was 3.8 days for galcanezumab 120 mg, 3.6 days for galcanezumab 240 mg, and 2.3 days for placebo. The mean differences versus placebo were statistically significant for both treatment groups (for 120 mg, mean difference = -1.56 days, 95 % CI, -2.37 to -0.75, *p* < 0.001; for 240 mg, mean difference = -1.33 days, 95 % CI, -2.14 to -0.52, *p* = 0.001).

#### Migraine headache days with prodromal symptoms

**Fig. 3:**
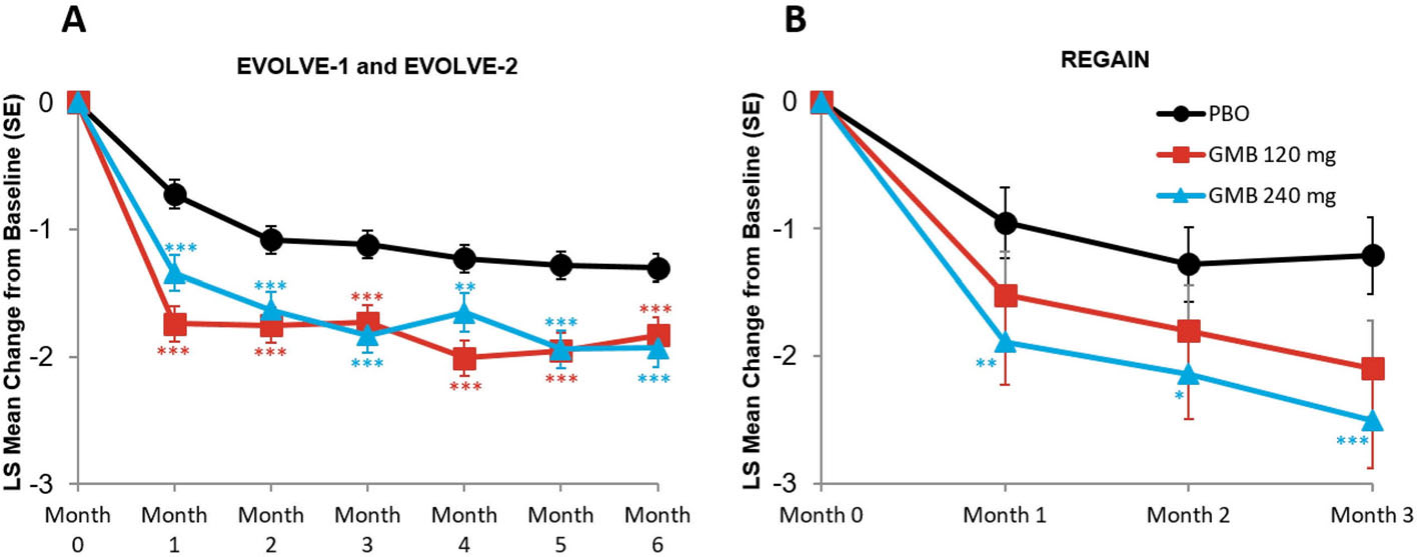
Monthly mean change in monthly migraine headache days with prodromal symptoms. *p≤0.05 vs PBO; **p≤0.01 vs PBO; ***p≤0.001 vs PBO. GMB, galcanezumab; LS,least squares; PBO, placebo; SE, standard error

The monthly mean change in monthly migraine headache days with prodromal symptoms are presented in Fig. 3A and B. In the episodic migraine studies, mean reduction from baseline in the number of monthly migraine headache days with prodromal symptoms across all 6 months was 1.8 days for galcanezumab 120 mg, 1.7 days for galcanezumab 240 mg, and 1.1 days for placebo. The mean differences versus placebo were statistically significant for both treatment groups (for 120 mg, mean difference = -0.71 days, 95 % CI, -0.96 to -0.47, *p* < 0.001; for 240 mg, mean difference = -0.60 days, 95 % CI, -0.84 to -0.35, *p* < 0.001). In the chronic migraine study, mean reduction from baseline in the number of monthly migraine headache days with prodromal symptoms across all 3 months was 1.8 days for galcanezumab 120 mg, 2.2 days for galcanezumab 240 mg, and 1.2 days for placebo. The mean differences versus placebo were statistically significant for both treatment groups (for 120 mg, mean difference = -0.66 days, 95 % CI, -1.29 to -0.02, *p* = 0.042; for 240 mg, mean difference = -1.03 days, 95 % CI, -1.67 to -0.40, *p* = 0.001).

#### Migraine headache days with aura

**Fig. 4:**
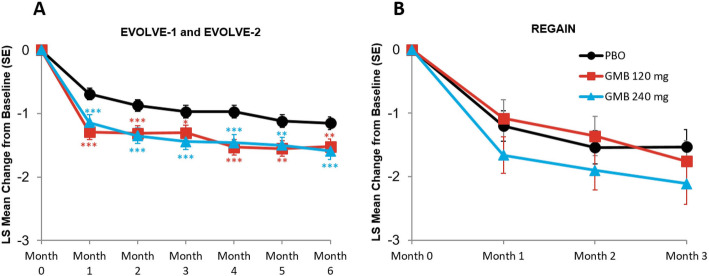
Monthly mean change in monthly migraine headache days with aura. *p≤0.05 vs PBO; **p≤0.01 vs PBO; ***p≤0.001 vs PBO. GMB, galcanezumab; LS, least squares; PBO, placebo;SE, standard error

The monthly mean change in monthly migraine headache days with aura are presented in Fig. 4A and B. In the episodic migraine studies, mean reduction from baseline in the number of monthly migraine headache days with aura across all 6 months was 1.4 days for galcanezumab 120 mg, 1.4 days for galcanezumab 240 mg, and 1.0 days for placebo. The mean differences versus placebo were statistically significant for both treatment groups (for 120 mg, mean difference = -0.46 days, 95 % CI, -0.67 to -0.24, *p* < 0.001; for 240 mg, mean difference = -0.45 days, 95 % CI, -0.67 to -0.24, *p* < 0.001). In the chronic migraine study, mean reduction from baseline in the number of monthly migraine headache days with aura across all 3 months was 1.4 days for galcanezumab 120 mg, 1.9 days for galcanezumab 240 mg, and 1.4 days for placebo. The mean differences versus placebo were not statistically significant for both treatment groups (for 120 mg, mean difference = 0.03 days, 95 % CI, -0.53 to 0.58, *p* = 0.922; for 240 mg, mean difference = -0.47 days, 95 % CI, -1.02 to 0.09, *p* = 0.098).

#### Proportion of remaining migraine headache days with nausea and vomiting

In the episodic migraine studies, among the three treatment groups, patients experienced nausea and vomiting on 41.2 % of the migraine headache days during the baseline period (Table 1). During the double-blind phase, the overall mean monthly reduction in the proportion of remaining migraine headache days with nausea and/or vomiting was 5.8 % for galcanezumab 120 mg and 6.8 % for galcanezumab 240 mg, which were significantly greater than the corresponding reduction of 2.5 % for the placebo group (*p* = 0.014 for galcanezumab 120 mg vs. placebo; *p* = 0.001 for galcanezumab 240 mg vs. placebo).

In the chronic migraine study, in the three treatment groups combined, patients experienced nausea and vomiting on 44.8 % of migraine headache days at baseline (Table 1). The overall mean monthly reduction across all 3 months in the percent of remaining migraine headache days with nausea and/or vomiting was significantly higher for galcanezumab 120 mg compared with the placebo group (7.3 % vs. 3.5 %, *p* = 0.01). The reduction from baseline in percent of migraine headache days for the 240 mg group was 6.2 % which was numerically greater compared with placebo although not statistically significant (*p* = 0.07).

#### Proportion of remaining migraine headache days with photophobia and phonophobia

During baseline, patients in the episodic migraine studies experienced photophobia and phonophobia on 73.9 % of migraine headache days. The overall mean monthly reduction from baseline across all 6 months in the percent of remaining migraine headache days with photophobia and phonophobia was 7.1 % for galcanezumab 120 mg and 8.1 % for galcanezumab 240 mg, which were significantly greater than the corresponding reduction of 3.2 % for the placebo group (*p* = 0.004 for galcanezumab 120 mg vs. placebo; *p* < 0.001 for galcanezumab 240 mg vs. placebo).

In the chronic migraine study, patients experienced photophobia and phonophobia on 74.8 % of migraine headache days on an average during the baseline period; however, the overall mean reduction in percent of remaining migraine headache days with photophobia and phonophobia was 2.5 % for galcanezumab 120 mg, and 3.2 % for galcanezumab 240 mg while the placebo group did not experience any meaningful reduction from baseline (0.8 %). The reductions for the two treatment groups, although numerically greater than placebo, were not statistically significant (*p* = 0.228 for galcanezumab 120 mg vs. placebo; *p* = 0.096 for galcanezumab 240 mg vs. placebo).

### Severity – reduction in moderate/severe migraine headache days, severe migraine headache days, severity of remaining migraine headache days

#### Reduction in moderate‐to‐severe migraine headache days

**Fig. 5:**
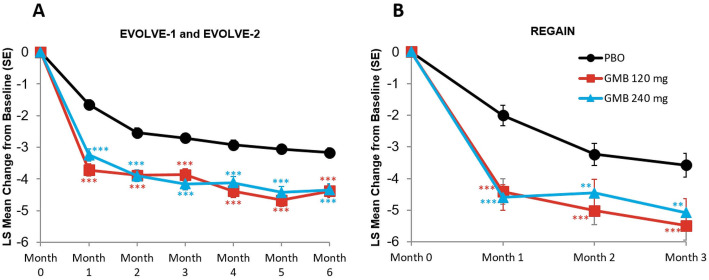
Monthly mean change in moderate-to-severemigraine headache days. **p≤0.01 vsPBO; ***p≤0.001 vs PBO. GMB,galcanezumab; LS, least squares; PBO, placebo; SE, standard error

The monthly mean change in moderate to severe migraine headache days are presented in Fig. 5A and B. In the episodic migraine studies, mean reduction from baseline in the number of moderate-to-severe migraine headache days across all 6 months was 4.2 days for galcanezumab 120 mg, 4.0 days for galcanezumab 240 mg, and 2.7 days for placebo. The mean differences versus placebo were statistically significant for both treatment groups (for 120 mg, mean difference = -1.48 days, 95 % CI, -1.80 to -1.16, *p* < 0.001; for 240 mg, mean difference = -1.36 days, 95 % CI, -1.68 to -1.04, *p* < 0.001).

In the chronic migraine study, mean reduction from baseline in the number of moderate-to-severe migraine headache days across all 3 months was 5.0 days for galcanezumab 120 mg, 4.7 days for galcanezumab 240 mg, and 2.9 days for placebo. The mean differences versus placebo were statistically significant for both treatment groups (for 120 mg, mean difference = -2.03 days, 95 % CI, -2.78 to -1.28, *p* < 0.001; for 240 mg, mean difference = -1.77 days, 95 % CI, -2.52 to -1.03, *p* < 0.001).

#### Reduction in severe migraine headache days

**Fig. 6:**
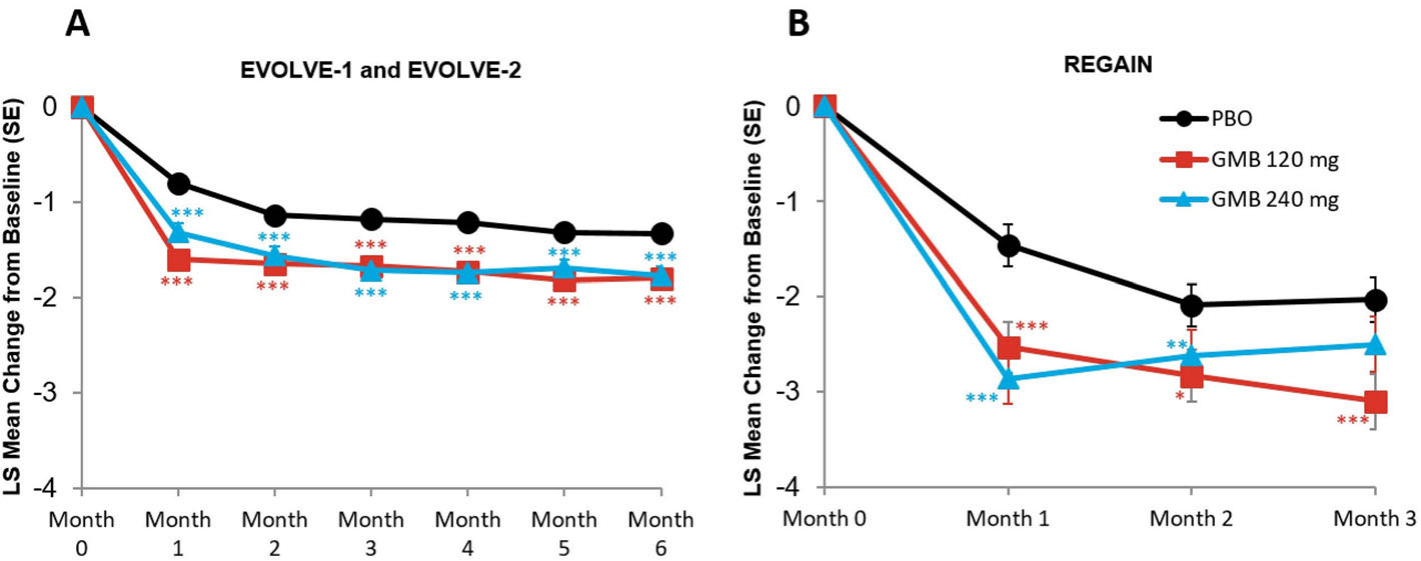
Monthly mean change in number of severe migraine headache days. *p≤0.05 vs PBO; **p≤0.01 vs PBO; ***p≤0.001 vs PBO. GMB, galcanezumab; LS, least squares; PBO, placebo;SE, standard error

The monthly mean change in number of severe migraine headache days are presented in Fig. 6A and B. In the episodic migraine studies, mean reduction from baseline in the number of severe migraine headache days across all 6 months was 1.7 days for galcanezumab 120 mg, 1.6 days for galcanezumab 240 mg, and 1.2 days for placebo. The mean differences versus placebo were statistically significant for both treatment groups (for 120 mg, mean difference = -0.55 days, 95 % CI, -0.70 to -0.39, *p* < 0.001; for 240 mg, mean difference = -0.47 days, 95 % CI, -0.62 to -0.31, *p* < 0.001).

In the chronic migraine study, mean reduction from baseline in the number of severe migraine headache days across all 3 months was 2.8 days for galcanezumab 120 mg, 2.7 days for galcanezumab 240 mg, and 1.9 days for placebo. The mean differences versus placebo were statistically significant for both treatment groups (for 120 mg, mean difference = -0.96 days, 95 % CI, -1.44 to -0.49, *p* < 0.001; for 240 mg, mean difference = -0.81 days, 95 % CI, -1.28 to -0.33, *p* < 0.001).

#### Reduction in severity of remaining migraine headache days

In the episodic migraine studies, the reduction from baseline in the mean severity of remaining migraine headache days across all 6 months was 0.2 for galcanezumab 120 mg, 0.2 for galcanezumab 240 mg, and 0.2 days for placebo. The mean differences versus placebo were statistically significant for both treatment groups (for 120 mg, mean difference = -0.04, 95 % CI, -0.08 to -0.00, *p* = 0.036; for 240 mg, mean difference = -0.06, 95 % CI, -0.10 to -0.02, *p* = 0.001).

In the chronic migraine study, the reduction from baseline in the mean severity of remaining migraine headache days across all 3 months was 0.2 for galcanezumab 120 mg, 0.2 for galcanezumab 240 mg, and 0.1 for placebo. The mean differences versus placebo were statistically significant for both treatment groups (for 120 mg and 240 mg, mean difference = -0.07, 95 % CI, -0.11 to -0.03, *p* < 0.001).

## Discussion

In the episodic and chronic migraine studies, both doses of galcanezumab demonstrated greater statistically significant improvements compared with placebo in the overall and the monthly reductions from baseline in the number of monthly migraine headache days. In the episodic migraine studies, the mean difference versus placebo in the overall (Months 1–6) mean reductions from baseline in the number of migraine headache days was statistically significant for both treatment groups (for 120 mg, mean difference = -1.84 days, 95 % CI, -2.25 to -1.42, *p* < 0.001; for 240 mg, mean difference = -1.88 days, 95 % CI, -2.30 to -1.47, *p* < 0.001) (EVOLVE-1 [18] and EVOLVE-2 [19]). In the chronic migraine study, the mean difference versus placebo in the overall (Months 1–3) mean reductions from baseline in the number of monthly migraine headache days was statistically significant for both treatment groups (for 120 mg, mean difference = -1.84 days, 95 % CI, -2.65 to -1.02, *p* < 0.001; for 240 mg, mean difference = -1.60 days, 95 % CI, -2.41 to -0.78, *p* < 0.001) (REGAIN [20]).

In the analyses reported here, galcanezumab was efficacious in reducing the severity and symptoms of migraine in patients with episodic and chronic migraine. Both the 120 mg and 240 mg doses of galcanezumab were superior to placebo in reducing the frequency of migraine headache days with associated symptoms such as nausea and/or vomiting, photophobia and phonophobia, and prodromal symptoms. In addition, among patients with episodic migraine, both doses of galcanezumab reduced the frequency of migraine headache days with aura, however, reduction in migraine headache days with aura was not statistically significant compared with placebo for either dose of galcanezumab in the chronic trial. This may have been due to the smaller sample size and shorter length of the chronic trial than in the two pooled episodic migraine trials. Moreover, although the magnitude of change for galcanezumab-treated patients was similar in patients with and without aura, there was a higher response in the placebo group for patients with aura than for patients without aura. Additionally, aura status was determined only by patient report and collected via ePRO in the prospective baseline period. As such, determination of aura status may have been influenced by a patient’s own interpretation of different symptoms. Consequently, limitations of interpretation of findings based upon self-reported aura have been highlighted by a number of authors [27, 28].

Since migraine-associated symptoms such as nausea, vomiting, and photophobia result in greater migraine-associated disability and disease burden [29, 30], it is important to reduce the number of migraine headache days with symptoms. In addition to the absolute reduction in number of migraine headache days with symptoms, patients would also benefit from fewer symptoms on days with headache. To explore this notion, we analyzed the change from baseline in the proportion of remaining migraine headache days with nausea and/or vomiting, and the proportion of remaining migraine headache days with photophobia and phonophobia. Among patients treated with galcanezumab, there was a greater reduction in the percent of migraine headache days with nausea and/or vomiting for both episodic and chronic migraine trials, and a reduction in the percent of migraine headache days with photophobia and phonophobia in the episodic migraine trials compared with patients treated with placebo. This suggests that treatment with galcanezumab results in fewer migraine headache days, and fewer days with symptoms among the migraine headache days that remain.

Migraine symptoms other than headache that occur during attacks such as nausea, photophobia, and phonophobia [5] are associated with significant disease burden [29–31]. People with migraine also self-report problems with taking acute and/or preventive oral migraine medications because of nausea and vomiting [31]. In the cross-sectional, observational Migraine in America Symptoms and Treatment (MAST) study, 64.9 % of all respondents (n = 7518) reported experiencing all 3 migraine symptoms of nausea, phonophobia, and photophobia [32]. Among patients who completed a 6-month follow-up assessment which included questions about their most bothersome migraine symptoms, patients reported that photophobia, nausea, and phonophobia were the most bothersome symptoms in up to 49.1 %, 28.1 %, and 22.8 % of patients, respectively [32]. A prospective cohort study using data from the American Migraine Prevalence and Prevention (AMPP) study showed that nausea seen in 43.7 % of patients with episodic migraine was associated with an increased risk of progression to chronic migraine in 3.4 % of patients [33]. A reduction in the associated symptoms of nausea and vomiting is particularly relevant since the presence of these symptoms is related to migraine-associated disability and increased burden of migraine [29].

In the study reported here, both doses of galcanezumab were superior to placebo in reducing the numbers of monthly moderate-to-severe migraine headache days. In the episodic and chronic migraine studies, both doses of galcanezumab were superior to placebo in reducing the overall and the number of monthly severe migraine headache days. In addition, although galcanezumab significantly reduced the mean severity of migraine headache days relative to placebo in both the episodic and chronic migraine patients, these differences were small, and it is unclear if they are clinically meaningful. Studies have shown that, with treatment, patients with migraine have a reduction in migraine days and moderate-to-severe headache days [16, 24]; however, there is a paucity of information on whether CGRP monoclonal antibodies reduce the most severe migraine headache days or most severe migraine attacks or only decrease the number of attacks and if these treatments lead to a reduction in migraine-associated symptoms such as nausea and/or vomiting, and photophobia and phonophobia. Recent post-hoc analysis of the same studies as reported here has also shown that treatment with galcanezumab 120 mg once monthly resulted in greater reduction in total pain burden in patients with episodic and chronic migraine compared to placebo [34]. The total pain burden is conceptualized as a measure of frequency of migraine headache days in a month, duration, and maximum severity of migraine headache on a given day, which, along with the results reported here, provide a more holistic view of the effect galcanezumab can have in patients with episodic or chronic migraine [34].

Some of the strengths of this study include the prospective nature of the randomized controlled studies, the large sample size, and the use of the ICHD-3 beta diagnostic criteria for the diagnosis of migraine. One of the limitations of the study was the method used to collect information related to aura in the trials. Patients self-reported the presence or absence of aura in the electronic patient-reported outcomes diary, rather than having a physician confirm the presence or absence of aura. Another limitation included the post-hoc nature of the analysis.

## Conclusions

Overall, this study demonstrates that, while treatment with galcanezumab decreases the frequency of migraine days compared with placebo, there is also a decrease in potentially disabling non-pain symptoms even on days when migraine is present.

## Data Availability

Data Sharing Statement **-** Lilly provides access to all individual participant data collected during the trial, after anonymization, with the exception of pharmacokinetic or genetic data. Data are available to request 6 months after the indication studied has been approved in the US and EU and after primary publication acceptance, whichever is later. No expiration date of data requests is currently set once data are made available. Access is provided after a proposal has been approved by an independent review committee identified for this purpose and after receipt of a signed data sharing agreement. Data and documents, including the study protocol, statistical analysis plan, clinical study report, blank or annotated case report forms, will be provided in a secure data sharing environment. For details on submitting a request, see the instructions provided at www.vivli.org.

## Abbreviations

CGRP: Calcitonin gene-related peptide
CI: Confidence interval
ePRO: Electronic patient-reported outcome
ICHD: International Classification of HeadacheDisorders
IHS: International Headache Society
LS: Least squares
MMRM: Mixed model repeated measures analysis
UI: Uncertainty interval
