# Correction to: Effect of galcanezumab on severity and symptoms of migraine in phase 3 trials in patients with episodic or chronic migraine

**DOI:** 10.1186/s10194-021-01307-6

**Published:** 2021-08-26

**Authors:** Michael Ament, Kathleen Day, Virginia L. Stauffer, Vladimir Skljarevski, Mallikarjuna Rettiganti, Eric Pearlman, Sheena K. Aurora

**Affiliations:** 1 Ament Headache Center, Denver, CO 80206 USA; 2 Eli Lilly and Company, Indianapolis, IN 46285 USA

**Correction to: J Headache Pain 22, 6 (2021)**

**https://doi.org/10.1186/s10194-021-01215-9**

Following the publication of the original article [1], the authors have notified us of a few mistakes in Table 1, marked with red below.

**Table 1:**
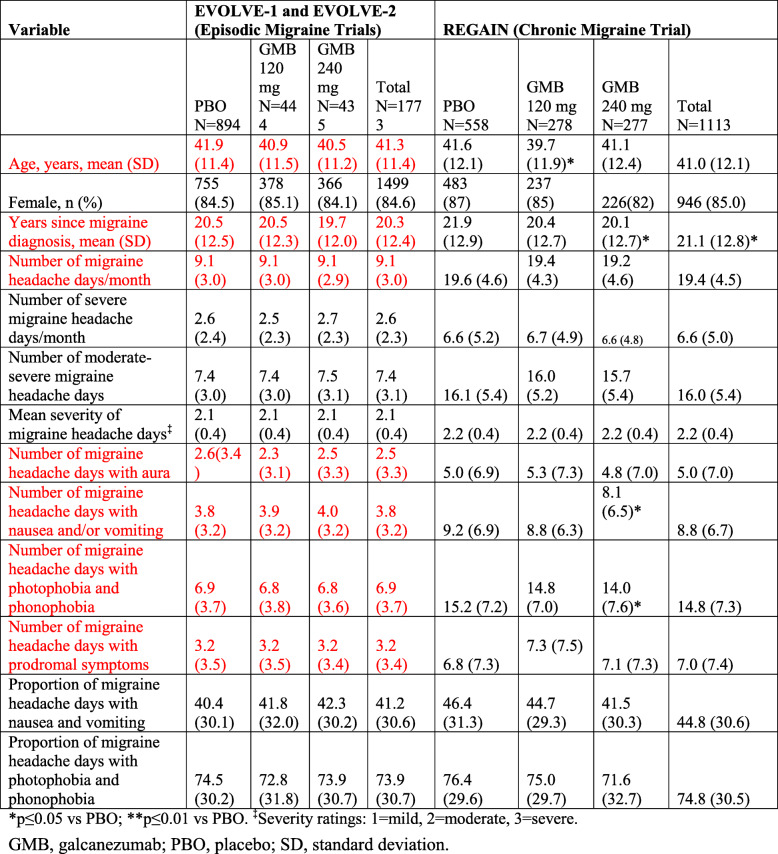
Baseline demographics and disease characteristics

*p ≤ 0.05 vs PBO; **p ≤ 0.01 vs PBO. ^‡^Severity ratings: 1 = mild, 2 = moderate, 3 = severe

*GMB* galcanezumab, *PBO* placebo, *SD* standard deviation
